# A methyltransferase LaeA regulates ganoderic acid biosynthesis in *Ganoderma lingzhi*

**DOI:** 10.3389/fmicb.2022.1025983

**Published:** 2022-10-14

**Authors:** Qin Luo, Na Li, Jun-Wei Xu

**Affiliations:** ^1^Faculty of Life Science and Technology, Kunming University of Science and Technology, Kunming, China; ^2^Faculty of Science, Kunming University of Science and Technology, Kunming, China

**Keywords:** ganoderma, ganoderic acids, regulator LaeA, biosynthesis, secondary metabolite

## Abstract

The methyltransferase LaeA is a global regulator involved in the biosynthesis of secondary metabolites by ascomycete fungi. However, little is known of its regulatory role in basidiomycete fungi. In this study, the *laeA* gene was identified in the basidiomycete *Ganoderma lingzhi* and its function in regulating the biosynthesis of anti-tumor ganoderic acids was evaluated. A *laeA* deletion (ΔlaeA) *Ganoderma* strain exhibited significantly reduced concentration of ganoderic acids. qRT-PCR analysis further revealed that the transcription levels of genes involved in the biosynthesis of ganoderic acids were drastically lower in the ΔlaeA strain. Moreover, deletion of *laeA* resulted in decreased accumulation of intermediates and abundances of asexual spores in liquid static culture of *G. lingzhi*. In contrast, constitutive overexpression of *laeA* resulted in increased concentration of ganoderic acids. These results demonstrate an essential role of LaeA in the regulation of ganoderic acid biosynthesis in *Ganoderma*.

## Introduction

*Ganoderm lingzhi* is a well-known medicinal fungus that has been used to improves health and prevent human diseases for over 2000 years (Bishop et al., 2015; Hsu and Cheng, 2018). Ganoderic acids (GAs) are lanosterol-type triterpenoids produced by *Ganoderma* that possess multiple bioactivities including anti-cancer, anti-inflammatory, antioxidant, and anti-HIV activities (Xu et al., 2010b; Ahmad et al., 2022). Moreover, different types of GAs exhibit distinct bioactivities. For example, ganoderic acid T (GA-T) induces apoptosis of lung cancer cells (Tang et al., 2006), and ganoderic acid Me (GA-Me) inhibits lung cancer metastasis (Chen et al., 2008).

**Figure 1:**
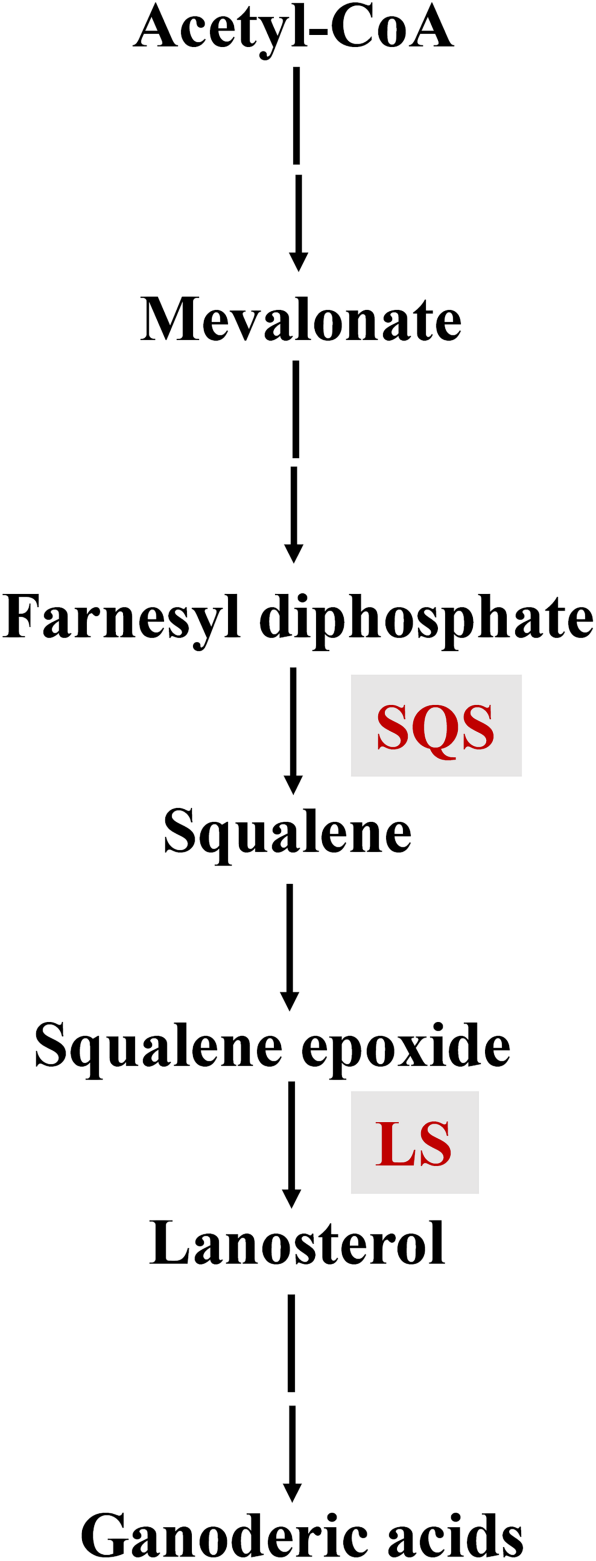
The biosynthetic pathway of ganoderic acids.

GAs are synthesized from the triterpene squalene, and the early biosynthetic steps are common for both GA and ergosterol pathways, including the sequential conversion of squalene to 2, 3-oxidosqualene, and lanosterol (Shi et al., 2010; Xu and Zhong, 2015). The downstream biosynthetic steps after lanosterol formation include several oxidation, reduction, and acetylation reactions (Xu et al., 2010a; Chen et al., 2012; Sun et al., 2021). During GA biosynthesis, squalene synthase (SQS) catalyzes the first step specific to triterpene synthesis, while lanosterol synthase (LS) is responsible for the formation of the lanostane skeletons of GAs (Figure 1).

Interest in regulating GA biosynthesis by *Ganoderma* has increased in recent years, due to their important pharmacological activities and commercial value. Environmental factors like heat stress, pH, and nitrogen sources all affect GA biosynthesis in *Ganoderma* (Zhao et al., 2011; Wu et al., 2016; Zhang et al., 2016). Further, signaling molecules like reactive oxygen species (ROS), cyclic adenosine monophosphate (cAMP), nitric oxide (NO), and Ca^2+^ participate in *Ganoderma* GA biosynthesis (Xu and Zhong, 2012; You et al., 2017; Ren et al., 2019; Liu et al., 2021). Moreover, the transcription factors AreA, PacC, and MADS1 are involved in regulating GA biosynthesis (Wu et al., 2016; Zhu et al., 2019; Meng et al., 2021). Besides, promoting sporulation was favorable to the biosynthesis of ganoderic acids in *G. lucidum* (Sun et al., 2021). Previous studies have indicated that the regulation of GA biosynthesis comprises a complex regulatory system. Further investigation of this system is needed to improve our understanding of GA biosynthesis regulation in *Ganoderma*.

The methyltransferase LaeA (loss of aflR expression-A) has been demonstrated to be involved in regulating the biosynthesis of numerous secondary metabolites in ascomycete fungi like *Aspergillus nidulans*, *Fusarium fujikuroi*, *Penicillium chrysogenum*, and *Trichoderma longibrachiatum* (Bok and Keller, 2004; Kosalkova et al., 2009; Wiemann et al., 2010; Shi et al., 2020). However, the function of LaeA has never been reported in other basidiomycete fungi, with the exception of *Coprinopsis cinerea*, in which the knockout of *laeA* improved coprinoferrin production (Tsunematsu et al., 2019). A LaeA ortholog has been identified in *Ganoderma* (Chen et al., 2012). However, it is currently unclear if and how LaeA influences GA biosynthesis in *Ganoderma*.

Here, we show that LaeA positively regulates GA biosynthesis in the basidiomycete *Ganoderma* for the first time. Targeted deletion of *laeA* significantly reduced GA production. Moreover, the expression of GA biosynthetic genes, accumulation of intermediates, and the abundance of asexual spores also decreased in the Δ*laeA Ganoderma*. Further analysis revealed that constitutive overexpression of *laeA* increased the production of GAs in *Ganoderma*.

## Materials and methods

### Strains and culture conditions

The strains *G. lingzhi* pJW-EXP-intron-opCas9 (Tu et al., 2021) and *G. lingzhi* CGMCC 5.616-1 (Sun et al., 2021) were maintained in our laboratory and used in this study. These strains were routinely maintained on potato dextrose agar and incubated at 30°C. The genotypes of the used *Ganoderma* strains are given in Supplementary Table S1. *Escherichia coli* JM109 was used for plasmid construction and cloning. Pre-culturing and liquid static fermentation of *G. lingzhi* mycelia were conducted as previously described (Xu et al., 2010a, 2012a). The fermentation medium for liquid static culture consisted of the following components (g/l): lactose, 35; KH_2_PO_4_·H_2_O, 1; MgSO_4_·7H_2_O, 0.5; peptone, 5; yeast extract, 5; and vitamin B1, 0.05. Asexual spores formed after 3 days of cultivation in liquid static condition.

### *In vitro* transcription of *laeA* sgRNAs and construction of the pJW-EXP-LaeA plasmid

Two sgRNA cassettes (Supplementary Data S1), including two *laeA* targeting sequences and a sgRNA sequence, were generated with a T7 promoter. The sequences were synthesized by Shanghai Sangon Led., Corp. (Shanghai, China). The two sgRNA cassettes were transcribed *in vitro* using a HiScribe™ T7 High Yield RNA Kit (NEB, Beijing, China) and purified using the RNA Clean and Concentration™ − 25 Kit (Zymo Research, Beijing, China).

The *laeA* sequence was amplified from *G. lingzhi* genomic DNA using the primers gpd-LaeA-F: 5′-ttcatccccctctcaacATGGCCATCGAATACGCCCG-3′ and ter-LaeA-R: 5′-ctctctgacccgctcat CTACAGGCGGCGCGCGT-3′. The amplified PCR product was fused with the plasmid pJW-EXP (Yu et al., 2014) that was digested with *Nhe*I using the ClonExpress MutiS one-step cloning kit (Vazyme, Nanjing, China) to produce the PJW-EXP-LaeA plasmid.

### Genetic transformation of *Ganoderma lingzhi* protoplasts and identification of transformants

PEG-mediated genetic transformation of *G. lingzhi* protoplasts was conducted as previously described (Xu and Zhong, 2015). Following the genetic transformation of the plasmid pJW-EXP-LaeA into the wild-type strain (monokaryotic CGMCC 5.616–1), LaeA transformants were screened on CYM selective plates containing 2 mg/l carboxin (Fei et al., 2019). Selective transformants were analyzed by PCR amplification of the fusion fragment containing the glyceraldehyde-3-phosphate dehydrogenase gene (*gpd*) promoter and *laeA* using the primers gpd-F: 5′-CGAGTGACGCAGGTGGTGAC-3′ and ter-R: 5′-GCAGTCGCACAATCTAGCCCT-3′. To screen the Δ*laeA* mutants, transformants were picked from CYM selective plates containing 250 mg/l hygromycin B after genetic transformation of *G. lingzhi* (the pJW-EXP-intron-opCas9 strain) protoplasts with the pJW-EXP-ophph plasmid (Tu et al., 2021) and the transcribed sgRNAs that targeted *laeA*. *laeA* was amplified from the genomic DNA of the control and mutant strains and then sequenced to confirm the gene deletion.

### Determination of mycelial growth, asexual spore numbers, GA contents, and the accumulation of squalene and lanosterol

Mycelial dry weight were measured using the gravimetric method. Briefly, mycelia were scraped from the surface of the liquid static culture and washed three times with distilled water. Mycelia were scraped from the surface of the liquid static culture and inoculated into H_2_O. The number of asexual spores (Xu et al., 2012b) was determined with a hemacytometer and expressed as the number of asexual spores per 1 cm^2^ (Zhang and Zhong, 2010; Sun et al., 2021). Total GAs and individual GAs, in addition to squalene and lanosterol, were extracted from *G. lingzhi* and determined using previously described methods (Zhou et al., 2014; Xu et al., 2019) and are shown in the Supplementary Data S2.

### Nucleic acid isolation

*Ganoderma lingzhi* mycelia were collected by filtration, washed with distilled water, frozen, and ground with liquid nitrogen. Genomic DNA was then extracted using the cetyltrimethylammonium bromide method (Saghai-Maroof et al., 1984), and RNA was extracted using TRIzol (Invitrogen, Carlsbad, CA, USA) according to the manufacturer’s protocol.

### Quantitative real time-PCR (qRT-PCR) analysis

Following RNA isolation, 1 μg of total RNA was treated with DNase I (Fermentas, Canada) and reverse-transcribed using the PrimeScript™ RT reagent kit (Takara, China). The transcription levels of the squalene synthase gene (*sqs*), lanosterol synthase gene (*ls*), *laeA*, and *gl25098* were then determined with the cDNA pools by qRT-PCR, as previously described (Zhang et al., 2017). The qRT-PCR primers used for amplification of *sqs*, *ls,* and *gl25098* were also previously described (Sun et al., 2021). In addition, the primers used to amplify *laeA* included: qRT-LaeA-F: 5′-CCCACTCCGATCATTACCTCTC-3′ and qRT-LaeA-R: 5′-GGTTTAGCCCGTTTTGTCTTTC-3′. The transcription levels of target genes were normalized to the levels of the internal reference, the 18S-rRNA gene. Gene expression from the control strain was defined as 1.0, and the transcription levels of genes from other strains were expressed as fold changes in comparison to control strain expression. Relative expression levels were calculated using the 2^ΔΔCt^ method.

### Sequence analysis

Amino acid sequence alignments were performed using Clustal W (Thompson et al., 2002). Phylogenetic tree was constructed with MEGA 7.0 using the neighbor-joining method with 1,000 bootstraps (Kumar et al., 2016).

### Statistical analysis

Data are presented as averages for three biological replicates, and the error bars indicate standard deviations from three replicates. Statistical analysis were performed using student’s t-test. Differences with value of *p* < 0.05 were considered statistically significant.

## Results

### Identification of a *laeA* ortholog in *Ganoderma lingzhi*

**Figure 2:**
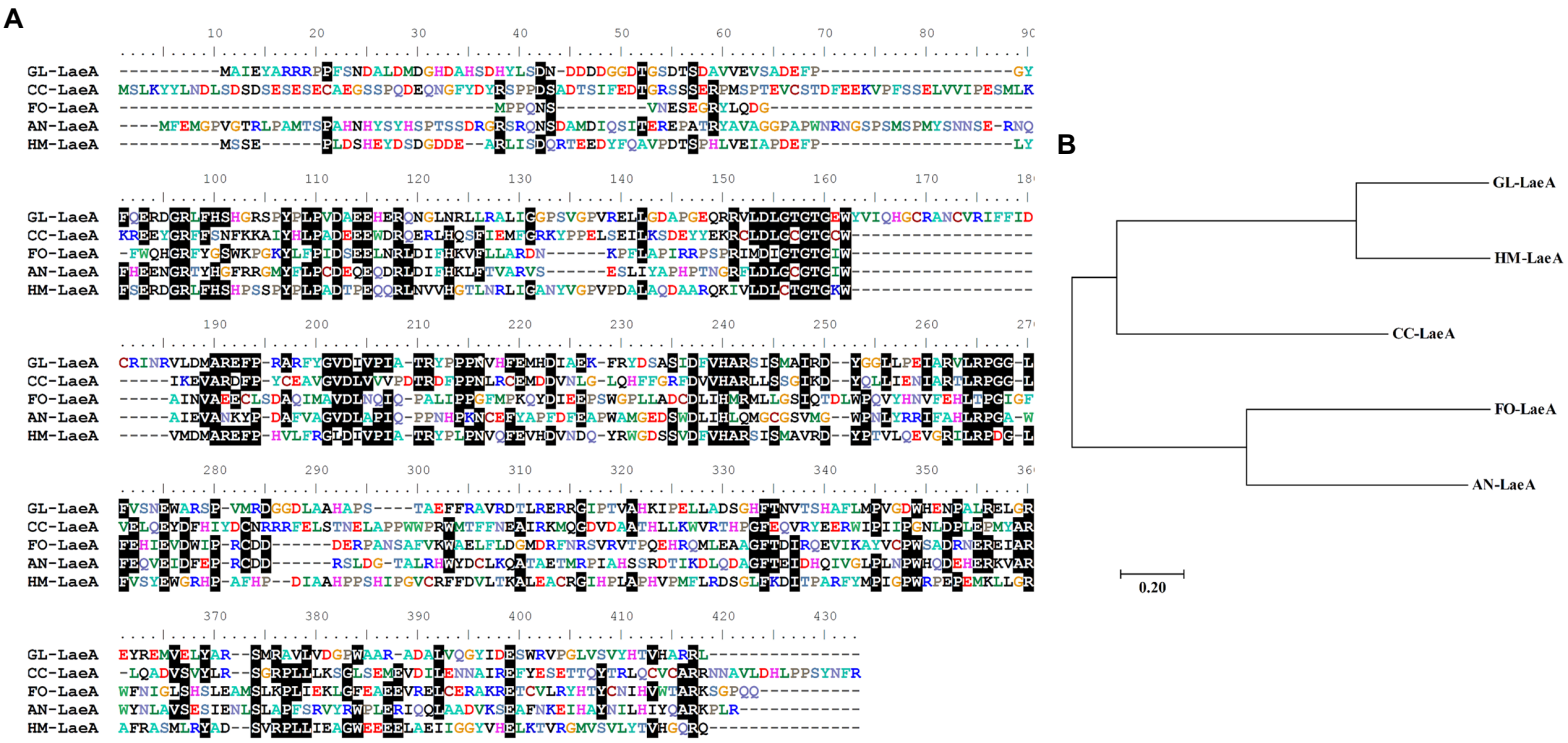
**(A)** Multiple alignment of amino acid sequences of GL-LaeA (*G. lingzhi*) and homologous proteins including CC-LaeA (*C. cinerea*, CC1G_00498), FO-LaeA (*F. oxysporum*, RKL38237.1), AN-LaeA (*A. nidulans*, C8VQG9.1), and HM-LaeA (*H. marmoreus*, RDB17513.1). Alignments were generated using ClustalW. **(B)** Phylogenetic reconstruction of amino acid sequences of LaeA from different strains. The tree was constructed using the neighbor-joining method with complete gap deletion in MEGA version 6.0.

To identify *laeA* ortholog encoded by *G. lingzhi*, its genome was queried using the LaeA amino acid sequence of *Hypsizygus marmoreus* (RDB17513). The protein encoding gene gl27879 that is hereafter referred to as LaeA was identified with 44% to RDB17543 and with a corresponding E value of <1.00E−80. *G. lingzhi laeA* is 1,379-bp long and has an open reading frame of 1,125-bp that encodes a protein of 375 amino acids. The amino acid residues in the 115–295 region of *G. lingzhi* LaeA encode an S-adenosylmethionine-dependent methyltransferase domain (Kadooka et al., 2020). Protein BLAST analysis revealed that *G. lingzhi* LaeA shares sequence identity with LaeA from *H. marmoreus, C. cinerea*, *Fusarium oxysporum*, and *A. nidulans*, (Figure 2A). Further, phylogenetic analysis indicated that *G. lingzhi* LaeA is more closely related to LaeA from basidiomycete*s* than to homologs in ascomycetes (Figure 2B).

### Deletion of *Ganoderma lingzhi laeA*

**Figure 3:**
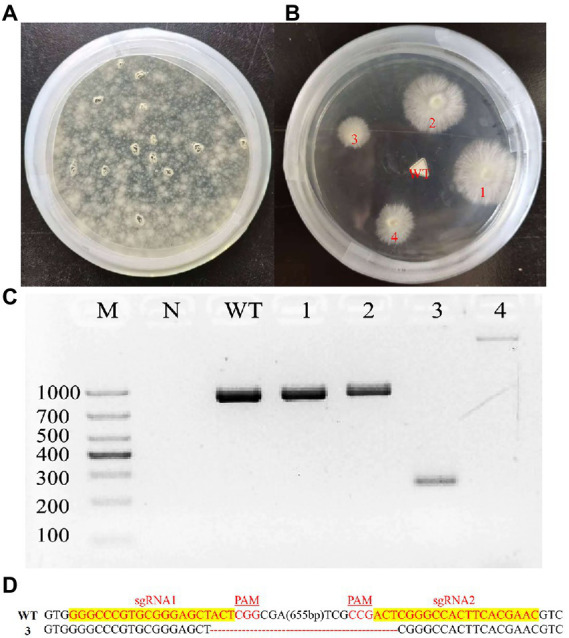
Deletion of *laeA* in *G. lingzhi*. **(A)** Screening and **(B)** re-selection of hygromycine resistant transformants after sgRNA1 and sgRNA2 targeted the *laeA* were delivered into the Cas9 strain. **(C)** Determination of *laeA* deletion in *G. lingzhi* transformants by genomic PCR. **(D)** TA-cloning of *laeA* deletions in selected transformant 3. The sgRNA-guiding sequences are highlighted in yellow. WT, wild-type strain; 3, the *laeA* deletion strain.

To delete the *laeA* of *G. lingzhi*, two *in vitro*-transcribed sgRNAs and the pJW-EXP-ophph plasmid were combined and transformed into *G. lingzhi* pJW-EXP-intron-opCas9 protoplasts using a PEG-mediated method (Liu et al., 2020; Tu et al., 2021). Numerous colonies were present on the selective CYM plates containing 250 mg/l hygromycin B (Figure 3A). Putative transformants were chosen from the selective CYM plates after three rounds of growth on nonselective CYM plates (Figure 3B). No evident morphological differences were observed when comparing the transformants and control strains (data not shown). The transformants were subsequently characterized by genomic PCR. Amplification of a clear band for *laeA* (950 bp) was observed in the control strain and transformants 1 and 2, while amplification of an approximately 250 bp band was observed for the transformant 3 (Figure 3C). Further, a > 1,000 bp amplicon was identified in transformant 4, indicating the presence of an insertion mutant. Sequence analysis of the PCR products indicated that the sequence between LaeA-sgRNA1 and LaeA-sgRNA2 was deleted, as expected in transformant 3 (Figure 3D). Thus, *laeA* was successfully deleted from *G. lingzhi.*

### Deletion of *laeA* reduced GA concentration by *Ganoderma lingzhi*

**Figure 4:**
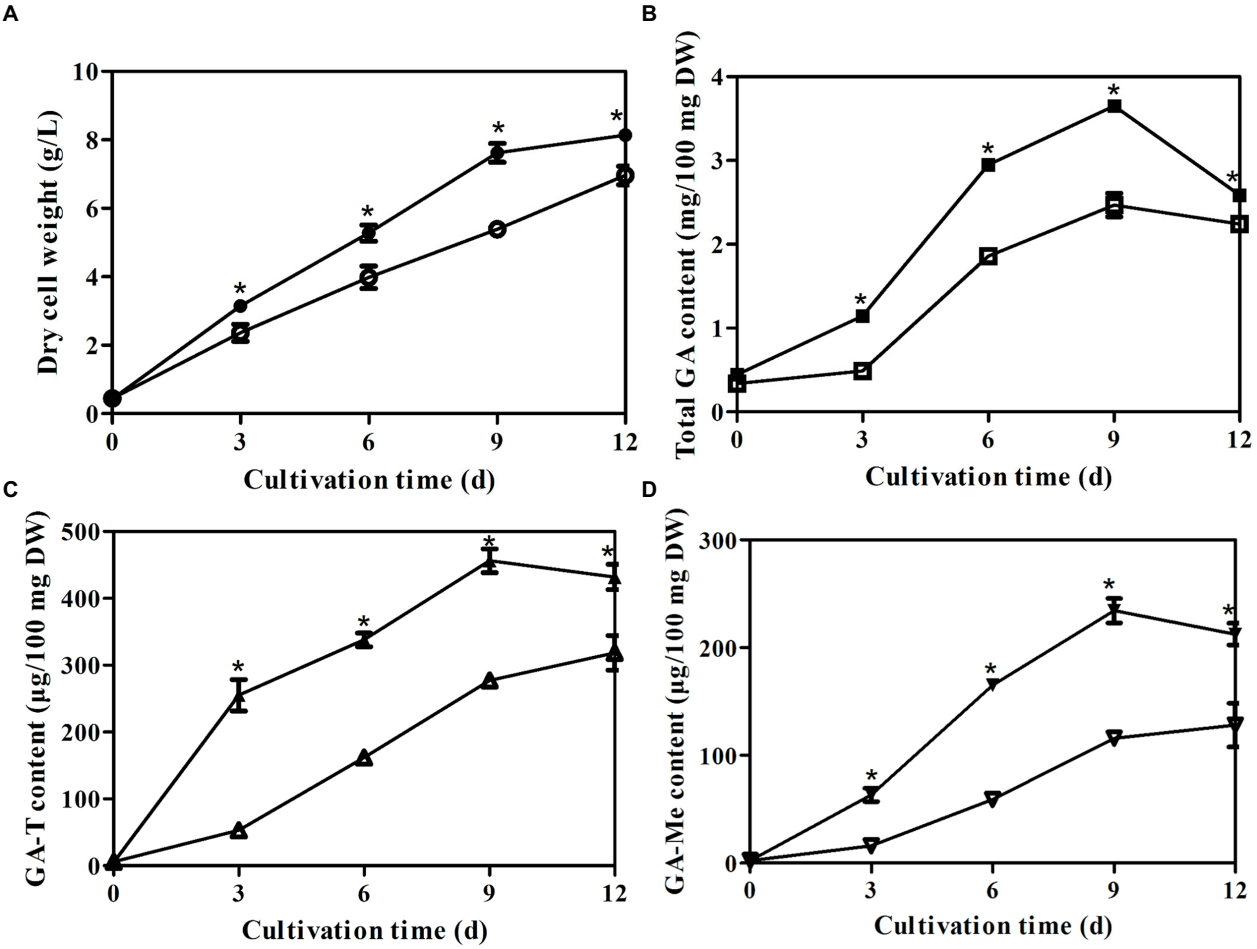
Temporal profiles of **(A)** mycelial growth **(A)** and **(B)** concentrations of total GAs, **(C)** GA-T and **(D)** GA-Me in liquid static culture of control (filled) and Δ*laeA* (open) strains.*a significantly different value compared to the control strain.

To analyze the effects of *laeA* deletion on GA production by *G. lingzhi*, the kinetics of mycelial growth, total GA concentration, and the concentrations of GA-T and GA-Me were determined in liquid static culture conditions. Mycelial growth and accumulation of GA exhibited similar trends in the control and Δ*laeA* strain (Figure 4). The maximum dry cell weights in the control and Δ*laeA* strains were 8.13 and 6.95 g/l on day 12, respectively (Figure 4A). Thus, the Δ*laeA* strain exhibited a decrease in biomass accumulation by 15%. GA-T and GA-Me are the major GA components of *G. lingzhi* mycelia (Xu et al., 2019). Temporal analysis of total GAs, GA-T, and GA-Me in both the control and Δ*laeA* strains is shown in Figures 4B–D. GA concentrations significantly increased and reached maximum values at day 9, followed by a slight decrease until the end of the fermentation. The maximum concentrations of total GAs, GA-T, and GA-Me in the Δ*laeA* strain were 2.46 mg, 277 μg, and 115 μg per 100 mg dry weight on day 9, 12 and 12, respectively, representing decreases of 67, 60, and 49% compared to values on day 9 for the control strain, respectively.

### Effects of *laeA* deletion on accumulation of intermediates and expression of GA biosynthesis genes

**Figure 5:**
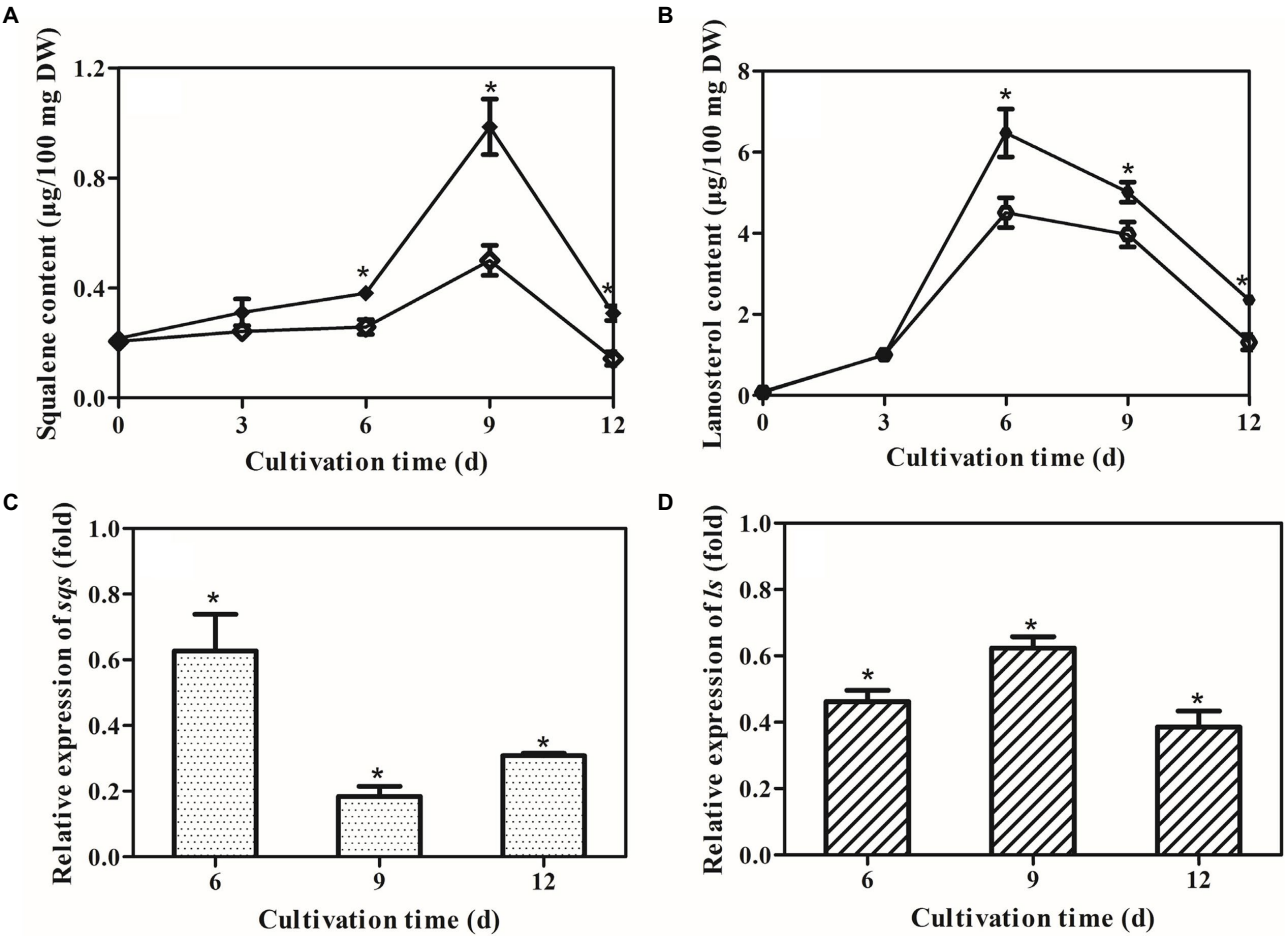
Effects of *laeA* deletion on intermediate accumulation and gene expression levels of *sqs* and *ls*. Accumulation of **(A)** squalene and **(B)** lanosterol in the control (filled) and Δ*laeA* (open) strains. Transcriptional levels of **(C)** *sqs* and **(D)** *ls* in the control and Δ*laeA* strains. Expression of genes in the control strain is defined as 1.0, and the expression levels in the Δ*laeA* strain are shown as fold changes compared to the reference.

Squalene and lanosterol are key intermediates in GA biosynthesis, and their accumulations were consequently determined in the control and Δ*laeA* strains. The concentrations of squalene and lanosterol increased until day 9 and day 6 (Figures 5A,B), respectively, and then decreased thereafter in both strains. The maximum squalene concentration observed in the Δ*laeA* strain was 0.5 μg/100 mg DW, representing a 0.51–time decrease in concentration compared to the control strain. The maximum lanosterol concentration in the Δ*laeA* strain was 4.5 μg/100 mg DW, representing a 67% decrease compared to the control strain. Thus, less intermediates accumulated in the Δ*laeA* strain compared to the control strain. The transcription levels of *sqs* in the Δ*laeA* strain were 62, 18, and 30% those of the control strain on days 6, 9, and 12, respectively (Figure 5C). *ls* transcription levels in the Δ*laeA* strain decreased to 46, 62, and 38% of the levels of the control strain on days 6, 9, and 12, respectively (Figure 5D).

### Deletion of *laeA* decreased asexual spore abundances in *Ganoderma lingzhi*

**Figure 6:**
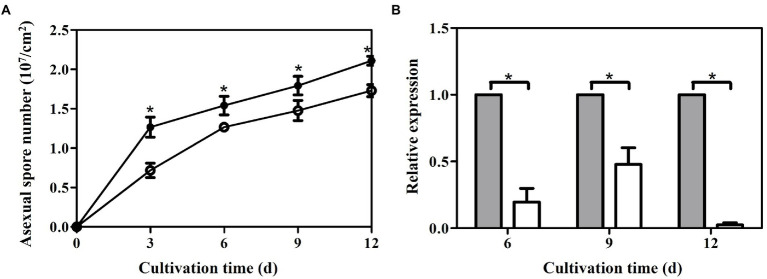
Temporal profiles of asexual spore numbers **(A)** and **(B)** transcription levels of *gl25098* during liquid static culture of the control (filled) and Δ*laeA* (open) strains. *, significant difference compared to the control value. The transcription levels of genes in the control strain are defined as 1.0, and the transcription levels of genes in the Δ*laeA* strain are expressed as fold changes relative to the control references.

The numbers of asexual spores were measured in both strains under liquid static culture condition. Temporal trends of asexual spore numbers were similar in the control (pJW-EXP-intron-opCas9 strain) and Δ*laeA* strains, wherein asexual spore numbers increased during fermentation and reached maximum values on day 12 (Figure 6A). The Δ*laeA* strain produced 1.72 × 10^7^ asexual spores per cm^2^ on day 12, representing 81% of that produced by the control strain. The transcription levels of the asexual sporulation specific gene *gl25098* (Sun et al., 2021) were also examined in the control and Δ*laeA* strains. The transcription levels of *gl25098* in the Δ*laeA* strain were 20, 47, and 3% of the levels in the control strain on days 6, 9, and 12, respectively (Figure 6B).

### LaeA overexpression in *Ganoderma lingzhi*

**Figure 7:**
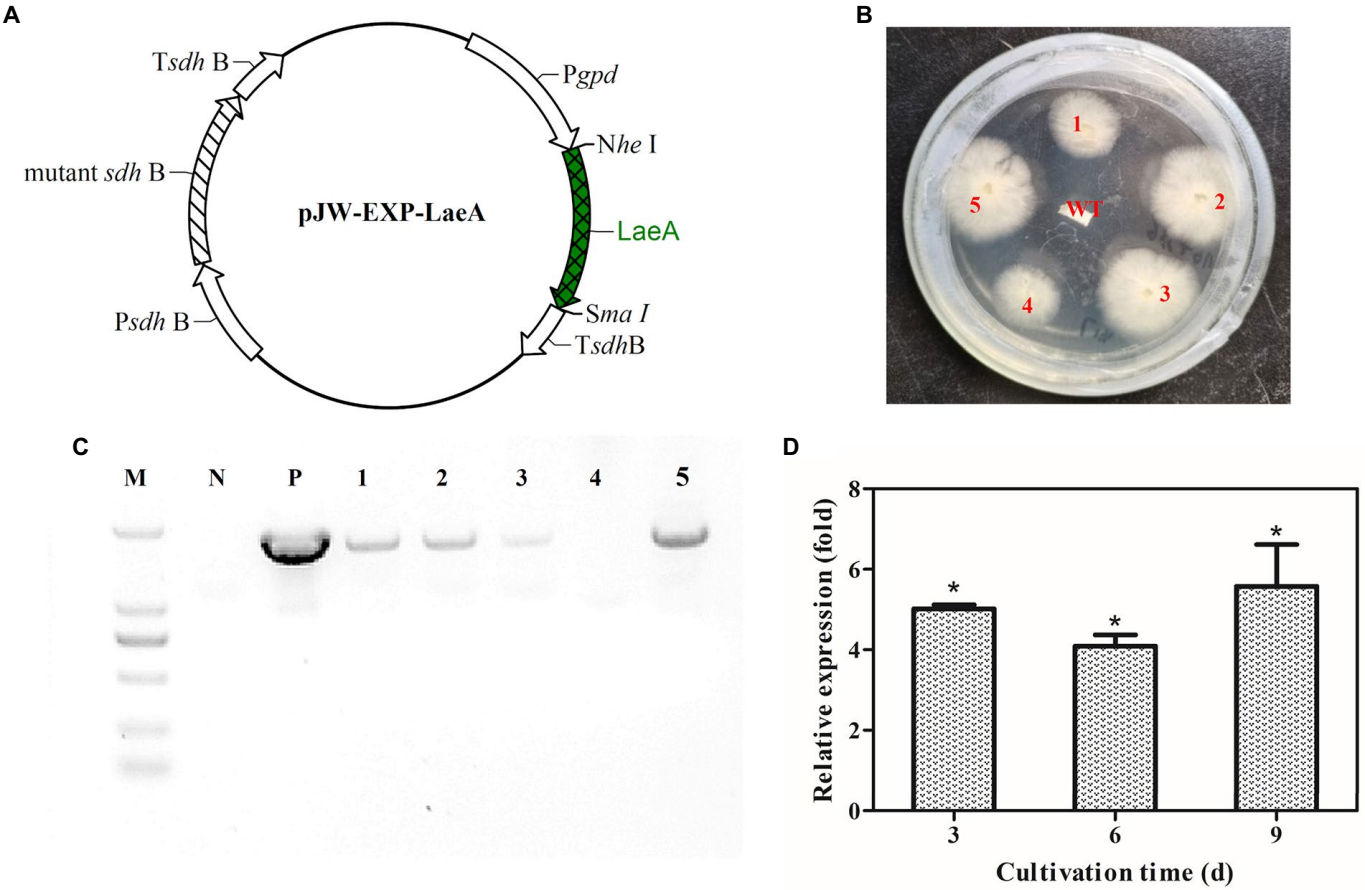
**(A)** Plasmid pJW-EXP-LaeA used for transformation of *G. lingzhi* protoplasts and identification of *laeA* overexpressing strains. **(B)** Selection of *laeA* transformants on selective CYM plates. **(C)** Amplification patterns obtained with primers for the fusion *gpd* promoter-*laeA* fragment in genomic DNA isolated from different strains. **(D)** Relative transcriptional levels of *laeA* in the WT and *laeA* transformant strains. M, DNA marker; P, positive control; WT, wild-type strain.

The plasmid pJW-Exp-LaeA (Figure 7A) was transformed into *G. lingzhi* (wild-type strain) protoplasts. Transformants were selected on CYM plates containing 2 mg/l carboxin after three rounds of growth on nonselective CYM plates (Figure 7B). The obtained transformants were confirmed with genomic PCR. Amplification yielded a clear band for the fused *gpd* promoter and the *laeA* fragment (1,670 bp) in the positive control and transformants 1, 2, 3, and 5 (Figure 7C). qRT-PCR analysis was then conducted to compare the transcription level of *laeA* in the mycelia of the wild type (WT) and the transformant 1 strains. *laeA* was overexpressed in transformant 1 under liquid static culture conditions. Further, the transcription levels of *laeA* in transformant 1 were 5.0-, 4.1-, and 5.5-fold higher than those of the WT strain on days 3, 6, and 9, respectively (Figure 7D).

### LaeA overexpression increased GA concentration in *Ganoderma lingzhi*

**Figure 8:**
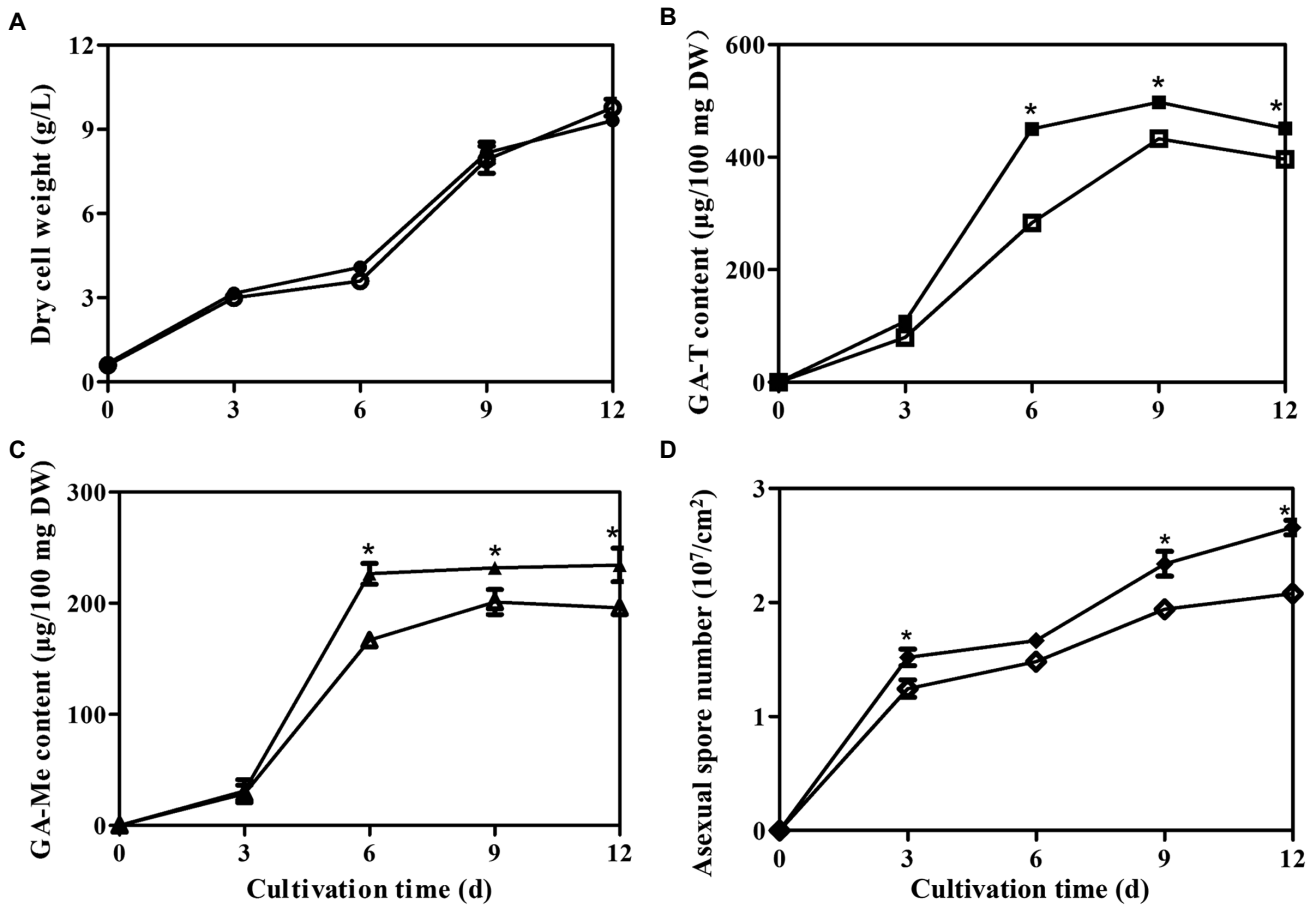
Temporal profiles of **(A)** mycelial growth, **(B)** Concentrations of GA-T and **(C)** GA-Me, and **(D)** asexual spore numbers in liquid static culture of wild-type *G. lingzhi* (open) and *laeA* overexpressing strains (filled).* significantly difference in values compared to the wild-type *G. lingzhi*.

The temporal trends of mycelial growth, concentrations of GA-Me and GA-T, and the abundances of asexual spores were evaluated in the *laeA* overexpressing and WT strains. Mycelial growth exhibited similar trends in both strains, with the maximum dry weights in the WT and *laeA* overexpressing strains being 9.31 and 9.75 g/l, respectively, under liquid static culture conditions (Figure 8A). Similar GA concentrations and asexual spore abundances were also observed for both strains (Figures 8B-D and Supplementary Figure S1). GA-T and GA-Me concentrations reached maximum levels on day 9 and declined on day 12, whereas the numbers of asexual spores increased during fermentation and reached maximum values at the end of fermentation. The maximum GA-T and GA-Me concentrations were 497 and 234 μg/100 mg DW in the *laeA* overexpressing strain, respectively, representing 1.25- and 1.20-fold higher values in the WT strain, respectively. In addition, the *laeA* overexpressing strain produced 2.70 × 10^7^ asexual spores per cm^2^ on day 12, representing a 25% higher abundance than the WT strain.

## Discussion

Here, the involvement of LaeA in GA biosynthesis regulation was investigated by gene deletion and overexpression experiments. Deletion of *laeA* led to reduced GA concentration, whereas overexpression of *laeA* led to increased GA concentration. These results indicated that LaeA is a positive regulator of GA biosynthesis in *Ganoderma*. Previous studies have shown that LaeA plays an important role in regulating some secondary metabolites in ascomycetes. For example, LaeA is a positive regulator of the biosynthesis of helvolic acid (Tsunematsu et al., 2019), penicillin (Kosalkova et al., 2009), bikaverin (Wiemann et al., 2010) and mycotoxins (Estiarte et al., 2016) in *Aspergillus fumigatus*, *P. chrysogenum*, *F. fujikuroi*, and *Alternaria alternata* CBS 116.329 strain, respectively. LaeA also negatively regulates the biosynthesis of the virulence factor dothistromin (Chettri and Bradshaw, 2016) and the mycotoxin alternariol (Estiarte et al., 2016) in *Dothistroma septosporum* and *A. alternata* ATCC 66981 strain, respectively. Tsunematsu et al. recently reported that the deletion of *laeA* resulted in increased production of the siderophore coprinoferrin in the basidiomycete *C. cinerea* (Tsunematsu et al., 2019). Thus, the identification and characterization of different *laeA* will facilitate a broader understanding of GA biosynthesis regulation in *Ganoderma*.

*sqs* and *ls* transcription levels were drastically decreased in the Δ*laeA* strain, suggesting LaeA regulated expression of GA biosynthesis genes. Similarly, deletion of *laeA* was shown to reduce the expression of the biosynthetic genes of sterigmatocystin (Bok and Keller, 2004) and bikaverin (Butchko et al., 2012) in *A. nidulans* and *Fusarium verticillioides*, respectively. It was hypothesized that LaeA could regulate the expression of genes involved in secondary metabolism by modifying fungal chromatin structure (Jain and Keller, 2013; Sarikaya-Bayram et al., 2015). However, the regulatory mechanism of GA biosynthesis by LaeA remains unclear and requires further investigation. The concentrations of the intermediates squalene and lanosterol were lower in the Δ*laeA* strain compared to the control strain, consistent with decreased production of GA. Decreased transcription levels of *sqs* and *ls* may lead to lower accumulations of squalene and lanosterol in the Δ*laeA* strains (Zhou et al., 2014; Zhang et al., 2017). The results from this study indicated that the decreased concentration of GAs in the Δ*laeA* strain could be attributed to the down-regulated expression of biosynthesis genes and decreased precursor concentrations. These results are consistent with previous observation of *Vitreoscilla* hemoglobin gene overexpression (Li et al., 2016a) and nitrogen limitation (Li et al., 2016b) in *G. lucidum*.

The Δ*laeA* strain accumulates fewer asexual spores than the control strain, and the numbers of asexual spores were higher in the *laeA* overexpressing strain than in the WT strain. qRT-PCR indicated that the expression levels of asexual sporulation-specific genes were lower in the Δ*laeA* strain. Thus, these results suggest that LaeA may be involved in the regulation of asexual sporulation in *Ganoderma*. Decreased asexual sporulation in *Aspergillus flavus* (Kale et al., 2008), *Alternaria alternate* (Takao et al., 2016), and *T. longibrachiatum* (Shi et al., 2020) *laeA* deletion strains had been previously documented. The influence of LaeA on asexual sporulation may be related to the expression of velvet family proteins in fungi (Jain and Keller, 2013; Aghcheh et al., 2014; Sarikaya-Bayram et al., 2015). Previous studies have shown that asexual spores accumulate higher levels of GAs than mycelia in liquid static culture of *G. lucidum* (Zhang and Zhong, 2010; Sun et al., 2021). The results of this study suggest that the decreased GA concentration in the Δ*laeA* strain may be at least partially related to decreased accumulation of asexual spores.

## Conclusion

In this study, the function of LaeA was investigated by gene deletion and overexpression in *G. lingzhi*. The results suggested that LaeA plays an important role in GA biosynthesis by regulating the expression of biosynthetic genes and asexual sporulation. These new insights help improve our understanding of the regulatory mechanisms of GA biosynthesis in *Ganoderma*.

## Data availability statement

The original contributions presented in the study are included in the article/Supplementary material, further inquiries can be directed to the corresponding authors.

## Author contributions

QL and NL: methodology, validation, investigation and writing—original draft preparation. J-WX: supervision, project administration, funding acquisition and writing—review and editing. All authors contributed to the article and approved the submitted version.

## Funding

This work was financially supported by the National Natural Science Foundation of China (No. 81860668) and the Yunnan Applied Basic Research Project (No. 2018FB065). J-WX also thanks to the Yunnan 10,000 Talents Plan-Young and Elite Talents Project.

## Conflict of interest

The authors declare that the research was conducted in the absence of any commercial or financial relationships that could be construed as a potential conflict of interest.

## Publisher’s note

All claims expressed in this article are solely those of the authors and do not necessarily represent those of their affiliated organizations, or those of the publisher, the editors and the reviewers. Any product that may be evaluated in this article, or claim that may be made by its manufacturer, is not guaranteed or endorsed by the publisher.

## References

[ref1] Aghcheh R. K. Nemeth Z. Atanasova L. Fekete E. Paholcsek M. Sandor E.. (2014). The VELVET a orthologue VEL1 of *Trichoderma reesei* regulates fungal development and is essential for cellulase gene expression. PLoS One 9, e112799. doi: 10.1371/journal.pone.0112799, PMID: 25386652 PMC4227869

[ref2] Ahmad M. F. Wahab S. Ahmad F. A. Ashraf S. A. Abullais S. S. Saad H. H. (2022). *Ganoderma lucidum*: a potential pleiotropic approach of ganoderic acids in health reinforcement and factors influencing their production. Fungal Biol. Rev. 39, 100–125. doi: 10.1016/j.fbr.2021.12.003

[ref3] Bishop K. S. Kao C. H. J. Xu Y. Glucina M. P. Paterson R. R. M. Ferguson L. R. (2015). From 2000 years of *Ganoderma lucidum* to recent developments in nutraceuticals. Phytochemistry 114, 56–65. doi: 10.1016/j.phytochem.2015.02.015, PMID: 25794896

[ref4] Bok J. W. Keller N. P. (2004). LaeA, a regulator of secondary metabolism in *aspergillus* spp. Eukaryot. Cell 3, 527–535. doi: 10.1128/ec.3.2.527-535.2004, PMID: 15075281 PMC387652

[ref5] Butchko R. A. E. Brown D. W. Busman M. Tudzynski B. Wiemann P. (2012). Lae1 regulates expression of multiple secondary metabolite gene clusters in *fusarium verticillioides*. Fungal Genet. Biol. 49, 602–612. doi: 10.1016/j.fgb.2012.06.003, PMID: 22713715

[ref6] Chen N. H. Liu J. W. Zhong J. J. (2008). Ganoderic acid me inhibits tumor invasion through down-regulating matrix metalloproteinases 2/9 gene expression. J. Pharmacol. Sci. 108, 212–216. doi: 10.1254/jphs.SC0080019, PMID: 18946196

[ref7] Chen S. L. Xu J. Liu C. Zhu Y. J. Nelson D. R. Zhou S. G.. (2012). Genome sequence of the model medicinal mushroom *Ganoderma lucidum*. Nat. Commun. 3:913. doi: 10.1038/ncomms1923, PMID: 22735441 PMC3621433

[ref8] Chettri P. Bradshaw R. E. (2016). LaeA negatively regulates dothistromin production in the pine needle pathogen *Dothistroma septosporum*. Fungal Genet. Biol. 97, 24–32. doi: 10.1016/j.fgb.2016.11.001, PMID: 27818262

[ref9] Estiarte N. Lawrence C. B. Sanchis V. Ramos A. J. Crespo-Sempere A. (2016). LaeA and VeA are involved in growth morphology, asexual development, and mycotoxin production in *Alternaria alternata*. Int. J. Food Microbiol. 238, 153–164. doi: 10.1016/j.ijfoodmicro.2016.09.003, PMID: 27642688

[ref10] Fei Y. Li N. Zhang D. H. Xu J. W. (2019). Increased production of ganoderic acids by overexpression of homologous farnesyl diphosphate synthase and kinetic modeling of ganoderic acid production in *Ganoderma lucidum*. Microb. Cell Factories 18:115. doi: 10.1186/s12934-019-1164-3, PMID: 31253150 PMC6599323

[ref11] Hsu K.-D. Cheng K.-C. (2018). From nutraceutical to clinical trial: frontiers in *Ganoderma* development. Appl. Microbiol. Biotechnol. 102, 9037–9051. doi: 10.1007/s00253-018-9326-5, PMID: 30182215

[ref12] Jain S. Keller N. (2013). Insights to fungal biology through LaeA sleuthing. Fungal Biol. Rev. 27, 51–59. doi: 10.1016/j.fbr.2013.05.004

[ref13] Kadooka C. Nakamura E. Mori K. Okutsu K. Yoshizaki Y. Takamine K.. (2020). LaeA controls citric acid production through regulation of the citrate exporter-encoding cexA gene in *aspergillus luchuensis* Mut. Kawachii. Appl. Environ. Microbiol. 86, e01950–19. doi: 10.1128/aem.01950-19, PMID: 31862728 PMC7028977

[ref14] Kale S. P. Milde L. Trapp M. K. Frisvad J. C. Keller N. P. Bok J. W. (2008). Requirement of LaeA for secondary metabolism and sclerotial production in *aspergillus flavus*. Fungal Genet. Biol. 45, 1422–1429. doi: 10.1016/j.fgb.2008.06.009, PMID: 18667168 PMC2845523

[ref15] Kosalkova K. Garcia-Estrada C. Ullan R. V. Godio R. P. Feltrer R. Teijeira F.. (2009). The global regulator LaeA controls penicillin biosynthesis, pigmentation and sporulation, but not roquefortine C synthesis in *Penicillium chrysogenum*. Biochimie 91, 214–225. doi: 10.1016/j.biochi.2008.09.004, PMID: 18952140

[ref16] Kumar S. Stecher G. Tamura K. (2016). MEGA7: molecular evolutionary genetics analysis version 7.0 for bigger datasets. Mol. Biol. Evol. 33, 1870–1874. doi: 10.1093/molbev/msw054, PMID: 27004904 PMC8210823

[ref17] Li H. J. He Y. L. Zhang D. H. Yue T. H. Jiang L. X. Li N.. (2016a). Enhancement of ganoderic acid production by constitutively expressing *Vitreoscilla* hemoglobin gene in *Ganoderma lucidum*. J. Biotechnol. 227, 35–40. doi: 10.1016/j.jbiotec.2016.04.017, PMID: 27080449

[ref18] Li H. J. Zhang D. H. Han L. L. Yu X. Y. Zhao P. Li T.. (2016b). Further improvement in ganoderic acid production in static liquid culture of *Ganoderma lucidum* by integrating nitrogen limitation and calcium ion addition. Bioprocess Biosyst. Eng. 39, 75–80. doi: 10.1007/s00449-015-1491-7, PMID: 26508324

[ref19] Liu K. Sun B. You H. Tu J. L. Yu X. Y. Zhao P.. (2020). Dual sgRNA-directed gene deletion in basidiomycete *Ganoderma lucidum* using the CRISPR/Cas9 system. Microb. Biotechnol. 13, 386–396. doi: 10.1111/1751-7915.13534, PMID: 31958883 PMC7017817

[ref20] Liu R. Zhu T. Yang T. Yang Z. Y. Ren A. Shi L.. (2021). Nitric oxide regulates ganoderic acid biosynthesis by the S-nitrosylation of aconitase under heat stress in *Ganoderma lucidum*. Environ. Microbiol. 23, 682–695. doi: 10.1111/1462-2920.15109, PMID: 32483888

[ref21] Meng L. Zhang S. Y. Chen B. Z. Bai X. R. Li Y. F. Yang J.. (2021). The MADS-box transcription factor GlMADS1 regulates secondary metabolism in *Ganoderma lucidum*. Mycologia 113, 12–19. doi: 10.1080/00275514.2020.1810515, PMID: 33085941

[ref22] Ren A. Shi L. Zhu J. Yu H. S. Jiang A. L. Zheng H. H.. (2019). Shedding light on the mechanisms underlying the environmental regulation of secondary metabolite ganoderic acid in *Ganoderma lucidum* using physiological and genetic methods. Fungal Genet. Biol. 128, 43–48. doi: 10.1016/j.fgb.2019.03.009, PMID: 30951869

[ref23] Saghai-Maroof M. A. Soliman K. M. Jorgensen R. A. Allard R. W. (1984). Ribosomal DNA spacer-length polymorphisms in barley Mendelian inheritance, chromosomal location, and population dynamics. Proc. Natl. Acad. Sci. U.S.A. 81, 8104–8108. 10.1073/pnas.81.24.8014 PMC392284 6096873

[ref24] Sarikaya-Bayram O. Palmer J. M. Keller N. Braus G. H. Bayram O. (2015). One Juliet and four Romeos: VeA and its methyltransferases. Front. Microbiol. 6, 1. doi: 10.3389/fmicb.2015.00001, PMID: 25653648 PMC4299510

[ref25] Shi J.-C. Shi W.-L. Zhou Y.-R. Chen X.-L. Zhang Y.-Z. Zhang X.. (2020). The putative methyltransferase TlLAE1 is involved in the regulation of peptaibols production in the biocontrol fungus *Trichoderma longibrachiatum* SMF2. Front. Microbiol. 11, 1267. doi: 10.3389/fmicb.2020.01267, PMID: 32612590 PMC7307461

[ref26] Shi L. A. Ren A. Mu D. S. Zhao M. W. (2010). Current progress in the study on biosynthesis and regulation of ganoderic acids. Appl. Microbiol. Biotechnol. 88, 1243–1251. doi: 10.1007/s00253-010-2871-1, PMID: 20859739

[ref27] Sun B. You H. Xu J. W. (2021). Enhancement of ganoderic acid production by promoting sporulation in a liquid static culture of *Ganoderma species*. J. Biotechnol. 328, 72–77. doi: 10.1016/j.jbiotec.2021.01.014, PMID: 33485862

[ref28] Takao K. Akagi Y. Tsuge T. Harimoto Y. Yamamoto M. Kodama M. (2016). The global regulator LaeA controls biosynthesis of host-specific toxins, pathogenicity and development of *Alternaria alternata* pathotypes. J. Gen. Plant Pathol. 82, 121–131. doi: 10.1007/s10327-016-0656-9

[ref29] Tang W. Liu H. W. Zhao W. M. Wei D. Z. Zhong J. J. (2006). Ganoderic acid T from *Ganoderma lucidum* mycelia induces mitochondria mediated apoptosis in lung cancer cells. Life Sci. 80, 205–211. doi: 10.1016/j.lfs.2006.09.001, PMID: 17007887

[ref30] Thompson J. D. Gibson T. J. Higgins D. G. (2002). Multiple sequence alignment using ClustalW and ClustalX. Curr. Protoc. Bioinform. 2, 2.3.1–2.3.22. doi: 10.1002/0471250953.bi0203s00 18792934

[ref31] Tsunematsu Y. Takanishi J. Asai S. Masuya T. Nakazawa T. Watanabe K. (2019). Genomic mushroom hunting decrypts coprinoferrin, a siderophore secondary metabolite vital to fungal cell development. Org. Lett. 21, 7582–7586. doi: 10.1021/acs.orglett.9b02861, PMID: 31496254

[ref32] Tu J. L. Bai X. Y. Xu Y. L. Li N. Xu J. W. (2021). Targeted gene insertion and replacement in the basidiomycete *Ganoderma lucidum* by inactivation of nonhomologous end joining using CRISPR/Cas9. Appl. Environ. Microbiol. 87:e0151021. doi: 10.1128/aem.01510-21, PMID: 34524900 PMC8579997

[ref33] Wiemann P. Brown D. W. Kleigrewe K. Bok J. W. Keller N. P. Humpf H. U.. (2010). FfVel1 and FfLae1, components of a velvet-like complex in *fusarium fujikuroi*, affect differentiation, secondary metabolism and virulence. Mol. Microbiol. 77, 972–994. doi: 10.1111/j.1365-2958.2010.07263.x, PMID: 20572938 PMC2989987

[ref34] Wu F. L. Zhang G. Ren A. Dang Z. H. Shi L. Jiang A. L.. (2016). The pH-responsive transcription factor PacC regulates mycelial growth, fruiting body development, and ganoderic acid biosynthesis in *Ganoderma lucidum*. Mycologia 108, 1104–1113. doi: 10.3852/16-079, PMID: 27760853

[ref35] Xu J. W. Xu N. Zhong J. J. (2012a). Enhancement of ganoderic acid accumulation by overexpression of an N-terminally truncated 3-hydroxy-3-methylglutaryl coenzyme a reductase gene in the basidiomycete *Ganoderma lucidum*. Appl. Environ. Microbiol. 78, 7968–7976. doi: 10.1128/aem.01263-12, PMID: 22941092 PMC3485969

[ref36] Xu J. W. Xu Y. N. Zhong J. J. (2010a). Production of individual ganoderic acids and expression of biosynthetic genes in liquid static and shaking cultures of *Ganoderma lucidum*. Appl. Microbiol. Biotechnol. 85, 941–948. doi: 10.1007/s00253-009-2106-5, PMID: 19578843

[ref37] Xu J. W. Yue T. H. Yu X. Y. Zhao P. Li T. Li N. (2019). Enhanced production of individual ganoderic acids by integrating *Vitreoscilla* haemoglobin expression and calcium ion induction in liquid static cultures of *Ganoderma lingzhi*. Microb. Biotechnol. 12, 1180–1187. doi: 10.1111/1751-7915.13381, PMID: 30821132 PMC6801144

[ref38] Xu J. W. Zhao W. Xu Y. N. Zhong J. J. (2012b). Isolation and analysis of differentially expressed genes during asexual sporulation in liquid static culture of *Ganoderma lucidum* by suppression subtractive hybridization. Mol. Biol. Rep. 39, 3603–3610. doi: 10.1007/s11033-011-1134-2, PMID: 21725848

[ref39] Xu J. W. Zhao W. Zhong J. J. (2010b). Biotechnological production and application of ganoderic acids. Appl. Microbiol. Biotechnol. 87, 457–466. doi: 10.1007/s00253-010-2576-5, PMID: 20437236

[ref40] Xu J. W. Zhong J. J. (2015). Genetic engineering of *Ganoderma lucidum* for the efficient production of ganoderic acids. Bioengineered 6, 357–360. doi: 10.1080/21655979.2015.1119341, PMID: 26588475 PMC4825833

[ref41] Xu Y. N. Zhong J. J. (2012). Impacts of calcium signal transduction on the fermentation production of antitumor ganoderic acids by medicinal mushroom *Ganoderma lucidum*. Biotechnol. Adv. 30, 1301–1308. doi: 10.1016/j.biotechadv.2011.10.001, PMID: 22036615

[ref42] You B. J. Tien N. Lee M. H. Bao B. Y. Wu Y. S. Hu T. C.. (2017). Induction of apoptosis and ganoderic acid biosynthesis by cAMP signaling in *Ganoderma lucidum*. Sci. Rep. 7:318. doi: 10.1038/s41598-017-00281-x, PMID: 28336949 PMC5428012

[ref43] Yu X. Y. Ji S. L. He Y. L. Ren M. F. Xu J. W. (2014). Development of an expression plasmid and its use in genetic manipulation of *Lingzhi* or *Reishi* medicinal mushroom, *Ganoderma lucidum* (higher basidiomycetes). Int. J. Med. Mushrooms 16, 161–168. doi: 10.1615/IntJMedMushr.v16.i2.60 24941037

[ref44] Zhang D. H. Li N. Yu X. Y. Zhao P. Li T. Xu J. W. (2017). Overexpression of the homologous lanosterol synthase gene in ganoderic acid biosynthesis in *Ganoderma lingzhi*. Phytochemistry 134, 46–53. doi: 10.1016/j.phytochem.2016.11.006, PMID: 27894599

[ref45] Zhang W. X. Zhong J. J. (2010). Effect of oxygen concentration in gas phase on sporulation and individual ganoderic acids accumulation in liquid static culture of *Ganoderma lucidum*. J. Biosci. Bioeng. 109, 37–40. doi: 10.1016/j.jbiosc.2009.06.024, PMID: 20129079

[ref46] Zhang X. Ren A. Li M. J. Cao P. F. Chen T. X. Zhang G.. (2016). Heat stress modulates mycelium growth, heat shock protein expression, ganoderic acid biosynthesis, and hyphal branching of *Ganoderma lucidum* via cytosolic Ca^2+^. Appl. Environ. Microbiol. 82, 4112–4125. doi: 10.1128/aem.01036-16, PMID: 27129961 PMC4959220

[ref47] Zhao W. Xu J. W. Zhong J. J. (2011). Enhanced production of ganoderic acids in static liquid culture of *Ganoderma lucidum* under nitrogen-limiting conditions. Bioresour. Technol. 102, 8185–8190. doi: 10.1016/j.biortech.2011.06.043, PMID: 21742489

[ref48] Zhou J. S. Ji S. L. Ren M. F. He Y. L. Jing X. R. Xu J. W. (2014). Enhanced accumulation of individual ganoderic acids in a submerged culture of *Ganoderma lucidum* by the overexpression of squalene synthase gene. Biochem. Eng. J. 90, 178–183. doi: 10.1016/j.bej.2014.06.008

[ref49] Zhu J. Sun Z. H. Shi D. K. Song S. Q. Lian L. D. Shi L.. (2019). Dual functions of AreA, a GATA transcription factor, on influencing ganoderic acid biosynthesis in *Ganoderma lucidum*. Environ. Microbiol. 21, 4166–4179. doi: 10.1111/1462-2920.14769, PMID: 31381838

