# Supplementary material for: A methyltransferase LaeA regulates ganoderic acid biosynthesis in Ganoderma lingzhi
Source: Front Microbiol. 2022 Oct 14;13:1025983. doi: 10.3389/fmicb.2022.1025983 (PMC9614229; doi:10.3389/fmicb.2022.1025983)
Supplement: Supplementary file 1 [file Data_Sheet_1.PDF]

# *Supplementary Material*

## 1. Supplementary Data S1

## >sgRNA1-LaeA gene cassette

TTCTAATACGACTCACTATAGGGCCCGTGCGGGAGCTACTGTTTTAGAGCTAGAAATAG
CAAGTTAAAATAAGGCTAGTCCGTTATCAACTTGAAAAAGTGGCACCGAGTCGGTGCTT
TT

## >sgRNA2-LaeA gene cassette

TTCTAATACGACTCACTATAGTTCGTGAAGTGGCCCGAGTGTTTTAGAGCTAGAAATAGC
AAGTTAAAATAAGGCTAGTCCGTTATCAACTTGAAAAAGTGGCACCGAGTCGGTGCTTT
T

Target sequence of *laeA* are underlined. T7 promoter sequence was highlighted in red-color font.

## 2. Supplementary Data S2

### 2.1 Extraction and analysis of individual ganoderic acids

For individual GAs, the dried mycelia (100 mg) were extracted by 75 % (v/v) ethanol (3 mL), ultrasonic treatment for 1 h (three times). The supernatants were dried at 50 $^{o}$C under vacuum and redissolved in 500 μl absolute ethanol for high performance liquid chromatography analysis. The elution was performed at a flow rate of 1.0 mL/min with a linear gradient of solvent A (methanol/acetic acid, 100:0.5, v/v) and solvent B (water). The gradient, starting at sample injection, was linear from 80 % to 100 % A in 20 min. Chromatographic peaks were identified by comparing the retention times and spectra against the known standards. The standards of GA-T and GA-Me were extracted and purified from mycelia with preparative liquid chromatography with purity over 99 %.

### 2.2 Extraction and analysis of squalene and lanosterol

Dried mycelia (50 mg) were saponified by 2 ml of 10 % (wt/vol) KOH–75% (v/v) ethanol solution at 50 $^{o}$C for 2 h. The mixture was extracted with 2 ml of hexane for three times. The hexane layer was collected and evaporated to dryness under N2. The residue was dissolved in 0.5 ml of acetonitrile for subsequent high-pressure liquid chromatography analysis. Chromatographic peaks were identified by comparing the retention times and spectra against the standards of squalene (⩾98%, Sigma) and lanosterol (⩾97%, Sigma, St. Louis, MO).

**Supplementary Figure S1**

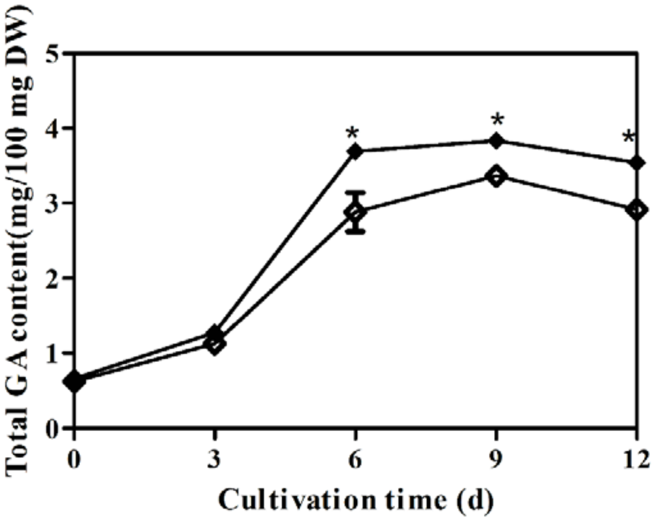


Figure S1. Temporal profiles of concentration of total GA in liquid static culture of wild-type *G. lingzhi* (open) and *laeA* overexpressing strains (filled).* significantly difference in values compared to the wild-type *G. lingzhi*.

**Supplementary Table S1**

Table S1. *Ganoderma* strains used in this study.

| Strain | Description | Reference |
|---|---|---|
| Wild-type | *Ganoderma* CGMCC 5.616-1 | Sun et al., 2021 |
| pJW-EXP-intron-opCas9 | opCas9 in *Ganoderma* | Tu et al., 2021 |
| Δ*laeA* | Δ *laeA* in pJW-EXP-intron-opCas9 | This study |
| *laeA* overexpressing | Overexpression of *laeA* in *Ganoderma* | This study |
